# The COVID-19 Pandemic and Global Food Security

**DOI:** 10.3389/fvets.2020.578508

**Published:** 2020-11-10

**Authors:** Fernando O. Mardones, Karl M. Rich, Lisa A. Boden, Andrea I. Moreno-Switt, Marisa L. Caipo, Natalia Zimin-Veselkoff, Abdulaziz M. Alateeqi, Isabelle Baltenweck

**Affiliations:** ^1^School of Veterinary Medicine, Pontifical Catholic University, Santiago, Chile; ^2^Department of Pediatric Infectious Diseases and Immunology, School of Medicine, Pontifical Catholic University, Santiago, Chile; ^3^International Livestock Research Institute, West Africa Regional Office, Dakar, Senegal; ^4^Global Academy of Agriculture and Food Security, The Royal (Dick) School of Veterinary Studies, The Roslin Institute, The University of Edinburgh, Edinburgh, United Kingdom; ^5^Millennium Initiative on Collaborative Research on Bacterial Resistance (MICROB-R), Santiago, Chile; ^6^Food and Agriculture Organization of the United Nations, FAO Regional Office for Latin America and the Caribbean, Santiago, Chile; ^7^Kuwait Institute for Scientific Research, Kuwait City, Kuwait; ^8^International Livestock Research Institute, Nairobi, Kenya

**Keywords:** COVID-19, food security, One Health, Planetary Health, SARS-CoV-2, food safety, animal production

## Abstract

We present scientific perspectives on the impact of the COVID-19 pandemic and global food security. International organizations and current evidence based on other respiratory viruses suggests COVID-19 is not a food safety issue, i.e., there is no evidence associating food or food packaging with the transmission of the virus causing COVID-19 (SARS-CoV-2), yet an abundance of precaution for this exposure route seems appropriate. The pandemic, however, has had a dramatic impact on the food system, with direct and indirect consequences on lives and livelihoods of people, plants, and animals. Given the complexity of the system at risk, it is likely that some of these consequences are still to emerge over time. To date, the direct and indirect consequences of the pandemic have been substantial including restrictions on agricultural workers, planting, current and future harvests; shifts in agricultural livelihoods and food availability; food safety; plant and animal health and animal welfare; human nutrition and health; along with changes in public policies. All aspects are crucial to food security that would require “One Health” approaches as the concept may be able to manage risks in a cost-effective way with cross-sectoral, coordinated investments in human, environmental, and animal health. Like climate change, the effects of the COVID-19 pandemic will be most acutely felt by the poorest and most vulnerable countries and communities. Ultimately, to prepare for future outbreaks or threats to food systems, we must take into account the Sustainable Development Goals of the United Nations and a “Planetary Health” perspective.

## Introduction

Food security is central to the United Nations 2030 Agenda for Sustainable Development Goals (SDG), which aim to end poverty and protect the planet from environmental degradation (1). Framed around these SDGs, the concept of “Planetary Health” emphasizes the understanding that human health and human civilization depend on wealthy natural systems and their prudent stewardship (2, 3). In addition to existing environmental changes (e.g., droughts, floods, extensive wildfires, typhoons, sea-level rise, etc.) that have recently led to major food crises (4), the world is now experiencing the worst pandemic since the Spanish flu in 1918. SARS-CoV-2 is the causative agent of COVID-19, a zoonotic respiratory epidemic that has been declared by the World Health Organization (WHO) as a global public health emergency (5). At the time of this writing, almost 10 months after the first case was discovered, the SARS-CoV-2 virus has affected more than 30 million people in 188 countries, and caused more than 1 million deaths (6). Both the disease and the fear of disease have triggered substantial global economic and social impacts, along with restrictions on international travel imposed by most countries, the quarantining of millions of people, dramatic declines in the tourism and hospitality industries, and disruption of supply chains for food, medicines, and manufactured products (7). As noted by food safety authorities, there is no evidence as yet associating the consumption of contaminated food or contaminated food packaging as routes of transmission of SARS-CoV-2 (8), yet taking precautions for this exposure route seems necessary.

The Food and Agriculture Organization of the United Nations (18) states that COVID-19 affects agriculture in two relevant aspects: the supply and demand for food. These two aspects place food security at risk in many key aspects of the food system value chain. We present examples where COVID-19 can impact food security in the short-, medium- and long-term based, primarily, on literature searches conducted independently by all authors. Searches were retrieved from electronic databases including Web of Science and Pubmed through the use of multiple keyworks and expressions, for example, (COVID^*^ OR coronavirus OR Sars-CoV-2) AND (food OR safety OR security OR nutrition) AND (nutrition^*^ OR health^*^ OR policy OR policies) that were collected in a reference manager system. In addition, gray literature including reports from international organizations, governments and non-governmental organizations and news from media were included upon agreement from all authors. Searches were restricted to include publications from 1 January through 1 September 2020.

We also provide sustainable “One Health” pathways to action which address the multi-dimensional nature of zoonotic and food-borne disease challenges, and which move preparedness and contingency planning for future threats to the food systems. “One Health” is a concept “*to address a health threat at the human-animal-environment interface based on collaboration, communication, and coordination across all relevant sectors and disciplines, with the ultimate goal of achieving optimal health outcomes for both people and animals; a ‘One Health' approach is applicable at the subnational, national, regional, and global level*” (9). Ultimately, COVID-19 provides an opportunity to move toward a holistic “Planetary Health” approach, defined as “*the health of human civilization and the state of the natural systems on which it depends*” (2, 3).

## Impacts on Agricultural Livelihoods and Food Availability

COVID-19 has disrupted many activities in fisheries, livestock, agriculture, and their supply chains; with outbreaks that have closed numerous facilities worldwide (10, 11). The use of quarantines, bans, restrictions on the movement of goods and people as disease control measures has resulted in significant socio-economic repercussions for livelihoods especially for poor rural farmers, livestock keepers, and capture fisheries from developing nations (7). Estimates on the economic fallout brought by the COVID-19 pandemic indicate that over half a billion people may be pushed into poverty. Of these, communities in Sub-Saharan Africa, North Africa and the Middle East are expected to be the hardest hit (12). Particularly, central and Southern Asia and sub-Saharan Africa, home to 87% of the world's extreme poor, will see the largest increases in extreme poverty, with an additional 54 million and 24 million people, respectively, living below the international poverty line as a result of the pandemic (13). Small island development states (SIDS) that depend on food imports will also be impacted.^1^ These sanitary restrictions, often indispensable to reduce the spread of the virus, also cause the frequent disruption of both market chains and trade of agricultural and non-agricultural products, entailing major potential impacts on the segments of the population that rely on them to sustain their livelihoods and their food and nutrition security (14).

Movement restrictions have reduced the availability of migrant labor, interrupting some harvesting and agricultural activities, increasing levels of post-harvest losses due to reduced workforce, and delaying the delivery of fresh produce to various target markets (15). Some examples include affected coffee growers in Brazil and Colombia, mango producers in Pakistan, and livestock in the UK. Although primary production may not appear to have suffered as harshly, a particular challenge in the short term will be to provide access to food for those in the population that are taking strict sanitary measures, particularly those who have lost their jobs and/or those in urban areas in countries where movement controls have limited the volumes of food traded from rural areas (16).

COVID-19 related disruptions and trends have made importers to continue facing insecurity in freight pricing, capacity, and demand volume across many modes of transport.^2^ These restrictions may also impact agricultural input markets by increasing the costs of storage at port and reducing the availability of seeds and fertilizer.^3^ There may also be a negative effect on animal feed and the ingredients necessary for the preparation of food, in particular those dependent on imports for their availability. China and India are countries of origin for many primary ingredients for both food and non-food imports, such as active pharmaceutical ingredients (17). Reliance on small numbers of overseas markets and channels of distribution, offers little resilience in the face of disruptions caused by this pandemic. The FAO has recommended facilitating transport and economic access to productive inputs (seeds, fertilizers, feed, etc.), along with access to machinery and infrastructure to ensure food supplies (18). Agriculture Ministers from the G20, African Union, ASEAN countries and Latin America and Caribbean (LAC) have agreed to keep global food markets open and refrain from imposing new trade barriers to ensure food flows between countries.

Informal, low-paid, and migrant workers are already highly vulnerable to food insecurity, defined as “*unreliable physical, social, and economic access to sources of adequate and nutritious food that meets people's dietary needs and food preferences*” (19). As a result of COVID-19, many have lost their jobs (20) and received no state support, with no social protection nets to allay potential impacts on hunger. Women largely work in the informal sector and face significant income losses as well.^4^ This is of particular relevance in conflict-affected places which host their displaced populations (21, 22).

Other workers continue their activities in conditions rife with poverty and in overcrowded spaces, which greatly increases the risk of contracting the disease. Informal markets have been closed down in several African countries, even though these markets are essential to provide vital food and sales outlets for low income consumers and low-income farmers and traders respectively. In such places, night markets, farmers' markets and roadside stalls are not allowed to operate during the movement control order, and many vegetable truck drivers have stopped their services as well, due to restrictions on traffic and operating hours, which has affected the food production chain (23).

While FAO (18) has noted that LAC and international traders have enough stocks to feed their populations in the next months, looking toward the longer-term, challenges to international trade, farm financial stability, and transportation remain in place (24). To maintain the availability of basic foods, it is key to maintain the operation of agricultural farms, with special attention to small scale farmers, but without excluding larger ones. Supporting the transportation, processing and packaging of agricultural and fishery products, solving logistical problems of food value chains and guaranteeing the operation of retail outlets, markets and supermarkets are key measures to keep the regional food system alive.

## Impacts on Food Safety

The risk of COVID-19 exposure and transmission via contact with domestic food-producing animals such as chickens, ducks, other poultry, pigs, cattle, horses or sheep or through consumption of contaminated food or exposure to food packages is currently considered negligible (25). However, concerns have been raised about the risks of human exposure to COVID-19 through the consumption of aquatic animals, such as finfish, crustaceans, mollusks and amphibians (26). Beijing has recently recorded dozens of new cases, all linked to a major wholesale fresh food market, raising concerns about a resurgence of the disease through this route of transmission.

The SARS-CoV-2 virus cannot multiply in food and requires an animal or human host to multiply. Aerosol and fomite transmission of the virus is the primary route of transmission and the virus can remain viable and infectious in aerosols for hours and survives on surfaces for days (27). There is not current scientific evidence to suggest that the virus is transmitted by eating contaminated food (28) nor can the virus grow or multiply on the surface of food stored in a cupboard, fridge, or freezer (29). However, it is possible that food animals and their products, as with other surfaces, could become contaminated with SARS-CoV-2 when handled by infected people that may shed the virus. New data have shown that SARS-CoV-2, in certain environmental conditions (e.g., 21–23°C), could survive in plastic for up to 3 days, in stainless steel for 2 days, and in cardboard for 1-day (27); this representing a potential risk and emphasize the importance of handwashing and good hygiene. While COVID-infected individuals have reported gastrointestinal symptoms with some having viral RNA or live virus in feces (30), viral RNA has also been detected in sewage (31, 32), suggesting that fecal-oral transmission is another possible route for exposure.

**Figure 1:**
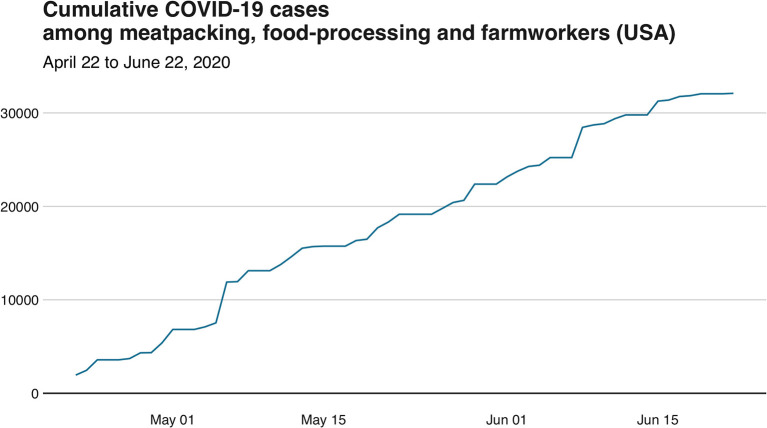
Cumulative number of total COVID-19 cases among meatpacking, food-processing and farmworkers in the US from April 22nd to June 22nd, 2020 (Source: Food and Environment Reporting Network at www.thefern.org).

Guidelines to mitigate risks of COVID-19 have been provided by the World Health Organization (WHO),^5^ the European Food Safety Authority (EFSA),^6^ the Food and Drug Administration (FDA),^7^ and the German Federal Institute for Risk Assessment (BfR).^8^ In addition, industry and relevant food business operators have taken important steps toward the reinforcement of measures for personal hygiene and food hygiene principles, in the form of refresher training, so as to help food workers reduce or eliminate the risk of contamination with the virus on food surfaces and food packaging materials. Nevertheless, the food industry has still been affected by facility closures (33) and numerous outbreaks (34). Data from the US indicates that there have been at least 32,000 COVID-19 cases related to employees of food systems, and most of these cases (~84%) have occurred in workers of meatpacking facilities (Figure 1). While not human COVID-19 cases have been attributed to the consumption or handling of raw meat or either food products from closed facilities were not recalled by FDA (35); environmental conditions of temperature and moisture at meat plants may facilitate SARS-CoV-2 indoor dispersal (36).

Meat processing plants, the meat packing industry and the poultry processing industry have been involved in superspreading events of COVID-19, particularly in the US, where, for example, the Smithfield plant in South Dakota accounts for 44% of all diagnoses in the state, making the largest single-source hot spot for the virus nationwide. In Minnesota, plant shutdowns have forced hog farmers to kill and dispose with the bodies of over 300,000 pigs. The ability to manage plant shutdowns downstream rests on the upstream capacity of the farm to manage more and larger animals, understood as providing enough space to house, feed and water them. Ideally, animals would be sent to a different abattoir to be processed, but this option is not necessarily available in every country, due to factors such as the lack of adequate means of transportation; unsuitable conditions of the alternate plant for the handling of a species or a certain animal size (e.g., incompatible equipment); not enough capacity for the accommodation of more animals; or a lack of personnel not unlike that affecting the original facility. Close to two million chickens had to be disposed of in Delaware due to a 50% shortage of personnel produced by the pandemic. In other words, there were simply not enough workers to process them, and there was insufficient space at their facility.

Reports of COVID-19 affecting food systems through contaminated imports has had negative consequences for industry, despite limited evidence of spread through this pathway. For example, China suspended imports from a pork plant in Germany and a chicken processor in the US, when a COVID-19 outbreak occurred (37, 38), while a beef unit in Brazil and a British pork plant voluntarily stopped exports to China after workers tested positive (39). While none of the outbreaks have been attributed to eating or handling contaminated food; in terms of current data, a recent pre-print showed that the titer of SARS-CoV-2 in artificially contaminated pieces of salmon, chicken and pork with 3 × 10^6^ TCDI_50_ (median tissue culture infectious dose) was stable at 4, −20°C, and −80°C (40). This is indicating that for some countries that appears to have eradicated the virus, there is a potential fear of re-emergence of COVID-19 by contaminated food and food packaging.

For decades, food producers have implemented food safety plans as pre-requisite programs, which include good hygienic practices. Despite this, there is still a lack of experience and evidence available on the risks of exposure and onward transmission of COVID-19 via food production workers. Risk assessments and implementation of effective interventions such as face masks and shields have been implemented to refine Food Safety Programs.^9^ More importantly, maintaining the safety and quality of food is critical, including the maintenance of testing for other known foodborne pathogens (e.g., *Listeria monocytogenes* and *Salmonella*), which could worsen the crisis in case of an outbreak (41). Countries and food industries should provide guidelines to stress any additional measures to ensure that the food chain is maintained, providing adequate and safe food supplies for consumers.

## Impacts on Plant and Animal Health and Animal Welfare

COVID-19 has compounded the impacts of other emerging and existing animal and plant disease threats, worsening health outcomes across different sectors and disproportionately affecting already marginalized populations (10, 11). For example, the regional and temporal clustering of COVID-19 outbreaks alongside climate change impacts (including severe weather such as flooding, droughts, heat waves, desertification, etc.), has exacerbated negative effects associated with the concurrent spread of other pests such as the desert locust plague (10, 11, 42). COVID-19 has reduced peoples' ability to conduct locust surveillance and control programs; the locusts have decimated food crops and forage in East Africa, India and Pakistan and this, in combination with severe economic crises in these countries, suggests that the negative health impacts of food insecurity may be felt long after the pandemic is over. Another example is the spread of the African Swine Fever virus (ASFv) in Asia (43). ASF does not pose any risk to human health but is a highly contagious (and fatal) viral disease of domestic and wild pigs. ASFv is responsible for serious production and economic losses and in 2019 reduced pig stocks in Southeast and East Asia, particularly China and Vietnam. Outbreaks remain ongoing in the region. Fighting and controlling pests and epidemics during a global pandemic is a potentially catastrophic combination that demands urgent responses in many countries, but also binds policymakers into making critical tradeoffs with the deployment of resources to address multi-faceted shocks. Thus, planning and preparation for epidemic prevention and control are essential.

## Impacts on Human Nutrition and Health

According to the estimates provided by the Global Report on Food Crises, in 2019, 135 million people were food insecure. More recent projections from the World Food Programme, however, indicate that this number may double to 265 million people in 2020, as a consequence of the effects the pandemic on the economy and the disruptions it has caused in supply chains (44). The pandemic brought by COVID-19 has put into display how the food, health and socioeconomic systems determining food outcomes are intricately interconnected. It has exposed again, how these systems currently operate in a manner that shields the richest and most powerful from many of the hardships of the pandemic (45).

The COVID-19 pandemic is creating worrying impacts on household incomes, food supply chains, health services, and schools (46). Moreover, strategies such as social distancing and hygiene measures like frequent handwashing are difficult to put into practice for the millions of people living in high density communities and whose housing is either precarious or insecure, with poor sanitation conditions and limited access to clean water. Many of those affected also face malnutrition and suffer from non-communicable diseases, and infectious diseases such as HIV/AIDS and tuberculosis (47). When the crisis began, an estimated 10.5 million children under the age of five suffered from wasting, 78 million children presented stunted growth, and 17 million were overweight, together with some 400 million women suffering from anemia (35). The present circumstances only worsen the difficulties already faced by a great number of families to access affordable and healthy diets.

The COVID-19 pandemic is causing an enormous impact on the nutrition status of the poorest and most vulnerable members of the population, and this impact should be regarded as a major concern. As demonstrated by the ecology of adversity and resilience, the health effects of substantial stressors, including inadequate nutrition, can lead to long-term effects (48). Indeed, poor-quality diets are linked to physical and also mental health (49). Recently, a recommended framework to sustain optimal nutrition from individual to global levels has been proposed to alleviate the impact of COVID-19 on nutrition and food security (50). FAO has also provided a set of guidelines on food systems and nutrition aimed at contributing to transform food systems and to promote sustainable food systems.^10^ Abundant literature exists on the relationship between food insecurity and poor health outcomes in children. Food insecurity has effects not only on human health but also on mental health. There is a higher risk of depression as well as suicidal ideation in adolescents, while chronic conditions, such as asthma, become more frequent. Iron deficiency, among other nutrient deficiencies, are known to be associated to impaired learning and decreased productivity in schoolchildren (51). Further evidence is required to understand the short and longer-term impact of COVID-19 on dietary intakes and resultant human nutrition and health. Low access to animal source foods, fruits, and vegetables has long term consequences, through poor child physical and cognitive development (52).

## Policies to Address Food Security: Current and Future Approaches

The post-pandemic phase may result in key changes within the food systems with emphasis on strengthening resilience to address the inequality of accessing healthy food (4). For example, locally produced food may be an opportunity for a new agri-food system that would reduce long-distance transportation and distribution by third parties with significant carbon footprints, although the evidence is mixed as to whether local production is always more “climate-friendly” (53). As in other conflicts, uncertainties about and/or an absence of governance, weakened institutions, changing donor funding priorities/involvement and diminished local research capacity constrain traditional opportunities for long-term contingency planning and access to and integration of local expertise that is essential for timely, evidence-based decision-making (54). Previous global outbreaks like Ebola (55) had adverse impacts on food and nutrition security, mostly for vulnerable populations including children and elderly, women, and the poor.

New or adapted policies will need to address tax and trade rules to continue the supply chain and adopting fiscal measures in case food prices abruptly increase (18). Currently, cash and in-kind transfers, new credit lines for strategic actors in the food chain, subsidies, loans and income support for families, distribution programs (e.g., food banks), and continuing school-feeding delivery for the most vulnerable and poorest people have been implemented to maintain trade and food supply chains while promoting social protection to ensure food access (56). Prices have declined for raw commodities such as wheat, vegetables, and other crops, yet consumers are often paying more for processed food products.

Last March, the United Nations allocated US$2 billion for a COVID-19 Global Humanitarian Response Plan, intended for agencies such as WHO, UNICEF, and the WFP to reach out to the most vulnerable communities and provide them with food, water and sanitation, and vaccinations, as well as testing materials COVID-19 and medical equipment (57).

### Recommended Actions

- **Accelerate progress toward the Sustainable Development Goals and strengthen local and global food systems by supporting local production, rural small-scale producer communities and backyard gardens in low middle-income countries**. Small scale farmers in Africa produce 72% of livestock derived foods (58). Such support will promote families and communities to feed themselves with diverse food and supporting the nearby urban areas with regular supplies. This approach has been proposed for Africa (59), where a strategic focus is required to provide key grassroot players in the food system, such as the communities of producers, fishers, pastoralists, indigenous peoples and others, with all the support and facilities they need.
- **Engage with consumers as well as producers to improve food system resilience to shocks**. Food systems are considered to be an important driver of climate change (60), with emergent impacts on the prevalence and distribution of novel infectious zoonotic and animal diseases as well as other direct impacts on greenhouse gas emissions and biodiversity loss (61). Understanding and influencing patterns of household consumption may play a powerful role in addressing resultant environmental and social impacts, as well as acting as a driver of reduced economic activity (62).
- **Identify unintended consequences and trade-offs of cross-sectoral interventions and policies to “future-proof” food systems**. For example, rewilding policies which aim to repair damaged ecosystems and restore degraded landscapes (63) may have indirect and unforeseen effects on human health and welfare including increases in traffic incidents and changes in disease dynamics (e.g., zoonosis) (64). Rampant deforestation, uncontrolled expansion and intensification of agriculture, and damaging activities such as drilling, mining, and infrastructure development are examples of unsustainable exploitation of wild nature and natural resources that have been recognized as main drivers for the incubation and transmission of diseases. Developing well designed rewilding plans demands a thorough understanding of interacting ecosystem processes and the socioeconomic context in which rewilding takes place.
- **Adopt risk-based approaches to target future interventions and policies to mitigate future shocks in the global food system and improve food security**. Despite the difficulty in predicting the impact of COVID-19, it is possible to determine the likely sources of transmission and forescast impacts on the most vulnerable. Risk-based approaches should focus on prevention strategies that are compatible with the local social context and a safe re-opening of the domestic economy with emphasis on food security. Relatively simple policies to encourage measures like the use of masks and handwashing stations to be put in place among informal markets would allow them to stay open and minimize risks to consumers and workers. More integrated approaches should use disease modeling or risk assessment frameworks as tools to support the decision-making process.
- **Increase/develop relevant research capacity and expertise through interdisciplinary training and research funding for scientists and practitioners**. Shocks to food systems, such as COVID-19 extend beyond a single-sector approach, demanding mobilization and integration of knowledge and skills across geographic, institutional and disciplinary boundaries. Sustainable food systems in the era of pandemics will require food production assistance and new tools, which include analyzing animal health and food safety through systematic approaches that will supply decision makers with significant added value (65).
- **Promote “One Health” and “Planetary Health” perspectives to cut across traditional domains to address the challenge posed by COVID-19**. The pandemic demonstrates our increasingly global, interdependent, and environmentally constrained societies. Broad integrated perspectives within the wider context of the SDGs are needed to properly address the impact of COVID-19, emerging infectious diseases and health threats on economics, international trade, politics, and inequality. In the future, our ability to prevent diseases and mitigate its impacts will depend on our competence to scale up action on the environment and avoid ruptures of ecological boundaries.

## Conclusions

The global COVID-19 pandemic, along with the implemented social distancing efforts intended to slow down its spread (66), have brought economies and food systems into disruption at a global and local scale, with wide ranging ramifications in terms of food security. Food insecurity is likely to lead into serious consequences in terms of public health.

Public health, which is largely how the COVID-19 response has been led and initially classified, appears to be insufficient to describe or deal with the consequences of this type of pandemic. Moreover, COVID-19 highlights that the concept of “One Health” covers more than just the emergence of an infectious disease, but also extends to food-related health outcomes. Ultimately, to prepare for future outbreaks or threats to food systems, one must to take into account the SDGs and “Planetary Health.” By doing so, we should be able to mitigate the impact of larger societal and political risks such as vulnerability, livelihoods, etc., and their interactions with the natural environment.

## Data Availability Statement

The raw data supporting the conclusions of this article will be made available by the authors, without undue reservation.

## Author Contributions

FM initially wrote the perspective and made Figure 1 based on COVID-19 cases at meatpacking facilities in the US. All authors listed have made a substantial, direct and intellectual contribution to the work, and approved it for publication.

## Conflict of Interest

The authors declare that the research was conducted in the absence of any commercial or financial relationships that could be construed as a potential conflict of interest.

## Footnotes

^1^https://www.theguardian.com/world/2020/jul/11/job-killer-of-the-century-economies-of-pacific-islands-face-collapse-over-covid-19

^2^https://www.freightos.com/freight-resources/coronavirus-updates/

^3^https://ihsmarkit.com/research-analysis/report-covid19-effects-on-the-fertilizer-industry.html

^4^https://blogs.worldbank.org/developmenttalk/covid-19-pivotal-moment-support-women-farmers

^5^https://www.who.int/publications/i/item/covid-19-and-food-safety-guidance-for-food-businesses

^6^https://www.efsa.europa.eu/en/news/coronavirus-no-evidence-food-source-or-transmission-route

^7^https://www.fda.gov/food/food-safety-during-emergencies/food-safety-and-coronavirus-disease-2019-covid-19

^8^https://www.bfr.bund.de/cm/349/can-the-new-type-of-coronavirus-be-transmitted-via-food-and-objects.pdf

^9^https://instituteforfoodsafety.cornell.edu/coronavirus-covid-19/background-info-covid-19/peer-reviewed-papers/

^10^http://www.fao.org/cfs/workingspace/workstreams/nutrition-workstream/e
